# Osteitis Condensans Ilii: An Uncommon Cause of Back Pain Masquerading as an Inflammatory Spondyloarthropathy

**DOI:** 10.7759/cureus.38935

**Published:** 2023-05-12

**Authors:** Henry Egbuchiem, Nkemputaife Onyechi, Onoja-Frederick Okwori, Akpezi Oshobe

**Affiliations:** 1 Internal Medicine, University Hospitals Geauga Medical Center, Cleveland, USA; 2 Internal Medicine, University Hospitals Cleveland Medical Center, Cleveland, USA; 3 Internal Medicine, Ashtabula County Medical Center, Cleveland, USA

**Keywords:** bone spurs, chronic inflammation, sclerosis, spondylitis, back pain

## Abstract

Osteitis condensans ilii (OCI), an uncommon cause of chronic back pain, remains a medical conundrum. The primary care physicians' limited awareness of this disease's clinical features, progression, diagnostic modality, and treatment principles creates a situation where the continuous use of numerous and sometimes unnecessary diagnostic tests leads to misdiagnosis of the etiology of chronic back pain and a rise in the cost of health care. Therefore, to further enhance the awareness of this disease, we present a case of osteitis condensans ilii masquerading as an unusual cause of chronic lower back pain in a postmenopausal female.

## Introduction

Osteitis condensans ilii (OCI) is a pathology of the sacroiliac bones in the pelvic cavity with an incidence of 0.9-2.5% [1]. Although rare, this bone disorder can commonly cause back or pelvic pain in women during pregnancy or puerperium. A symptomatic and asymptomatic variant characterizes the clinical course of osteitis condensans illi. OCI is responsible for about 1-2.5% of lower back pain when symptomatic [1]. The exact etiology of this condition is not yet known; however, it is postulated that OCI occurs due to mechanical stresses and imbalance across the sacroiliac joints [1,2]. Also, it is considered an incidental finding in many patients and often mimics inflammatory axial pathologies. The diagnosis depends on the presence of the characteristic sclerotic lesions on sacroiliac radiographs after excluding other conditions associated with back pain [3]. The overall management of this condition is conservative, with the use of physical therapy and analgesics. More importantly, in most cases, the prognosis is favorable [4]. Although OCI is an uncommon condition seen in women of childbearing age, we describe the case of OCI in a postmenopausal female with a concurrent history of back pain.

## Case presentation

A 67-year-old morbidly obese female, with a medical history of hypertension, hyperlipidemia, hypothyroidism, obstructive sleep apnea, and osteoarthritis of both knees with a left total knee replacement 15 years prior, presented to our clinic. She presented to the clinic because of progressive symptoms of chronic lower back pain, which started about five years ago. She rated the pain as 8/10 on the pain scale. The pain was also described as constant and located on the left side of her back. The pain was worse with lying down and radiating to her thigh but improved with sitting in a recliner, using heating pads, and taking non-steroidal anti-inflammatory drugs (NSAIDs). She also had occasional bilateral hand swelling (wrists and metacarpophalangeal joints) and morning stiffness lasting only a few minutes. She; however, denied chest pain, dyspnea, fever, cough, malaise, fatigue, night sweats, or recent weight loss. The patient also reported a history of back trauma as a teenager while hauling hay. The patient is gravida 1 para 1 (she has a 34-year-old son delivered via uncomplicated cesarean section). Surgical history was significant for laparoscopic cholecystectomy. She denies the use of alcohol, tobacco, and illicit substances. Her vital signs showed a temperature of 97.1°F, heart rate of 54 beats per minute, respiratory rate of 16 cycles per minute, blood pressure of 120/70 mmHg, and oxygen saturation of 100% on room air. She appeared alert and in no acute distress. Cardiovascular and pulmonary examinations were unremarkable.

The examination of her back showed symmetric posture and no erythema, edema, or swelling. Lumbar forward flexion was possible to about 90 degrees. Provocating pain symptoms caused a diminished extension and rotation to the right. The left side was normal. There was significant tenderness with palpation of the left sacroiliac joint area more than the right and some tenderness over the left gluteal and paraspinal muscles. Negative straight leg raise test and tenderness was over the left greater trochanteric bursa. The patient had a positive flexion, abduction, and external rotation (FABERS) test on the left. She had negative hip clicking and a negative resisted active straight leg raise. There was no pain with deep hip flexion. The patient had diminished sensation in the left lateral thigh. Her motor examination was 5/5 globally, gait normal. Initial laboratory studies showed the complete blood counts and metabolic panel within normal limits. Specific laboratory tests for inflammatory markers such as C-reactive protein, erythrocyte sedimentation rate, rheumatoid factors, and antinuclear antibody tests were negative. She also had a negative human leukocyte antigen (HLA) B27 genetic test. Other laboratory values for the patient are documented in Tables 1, 2 below.

**Table 1:** Complete blood count laboratory values.

Test	Value	Reference
White blood Cells	8.2/hpf	4-12/hpf
Hemoglobin	15.5 g/dL	14-16 g/dL
Hematocrit	46.4%	36-48%
Mean corpuscular volume	89 fL	80-100 fL
Mean corpuscular hemoglobin concentration	33.4 g/dL	32-36 g/dL
Platelets	309×10^9^/L	150-350×10^9^/L

**Table 2:** Laboratory study showing values of blood chemistry. LDL: low-density lipoprotein

Test	Value	Reference
Sodium	138 g/dL	135-145 g/dL
Potassium	3.7 g/dL	3.5-5.5 g/dL
Bicarbonate	28 mmol/L	22-32 mmol/L
Blood urea nitrogen	29 mg/dL	7-20 mg/dL
Creatinine	1.11 mg/dL	0.6-1.1 mg/dL
Cholesterol	179 mg/dL	<200 mg/dL
LDL	101 mg/dL	<100 mg/dL
Triglycerides	168 mg/dL	<150 mg/dL

**Figure 1:**
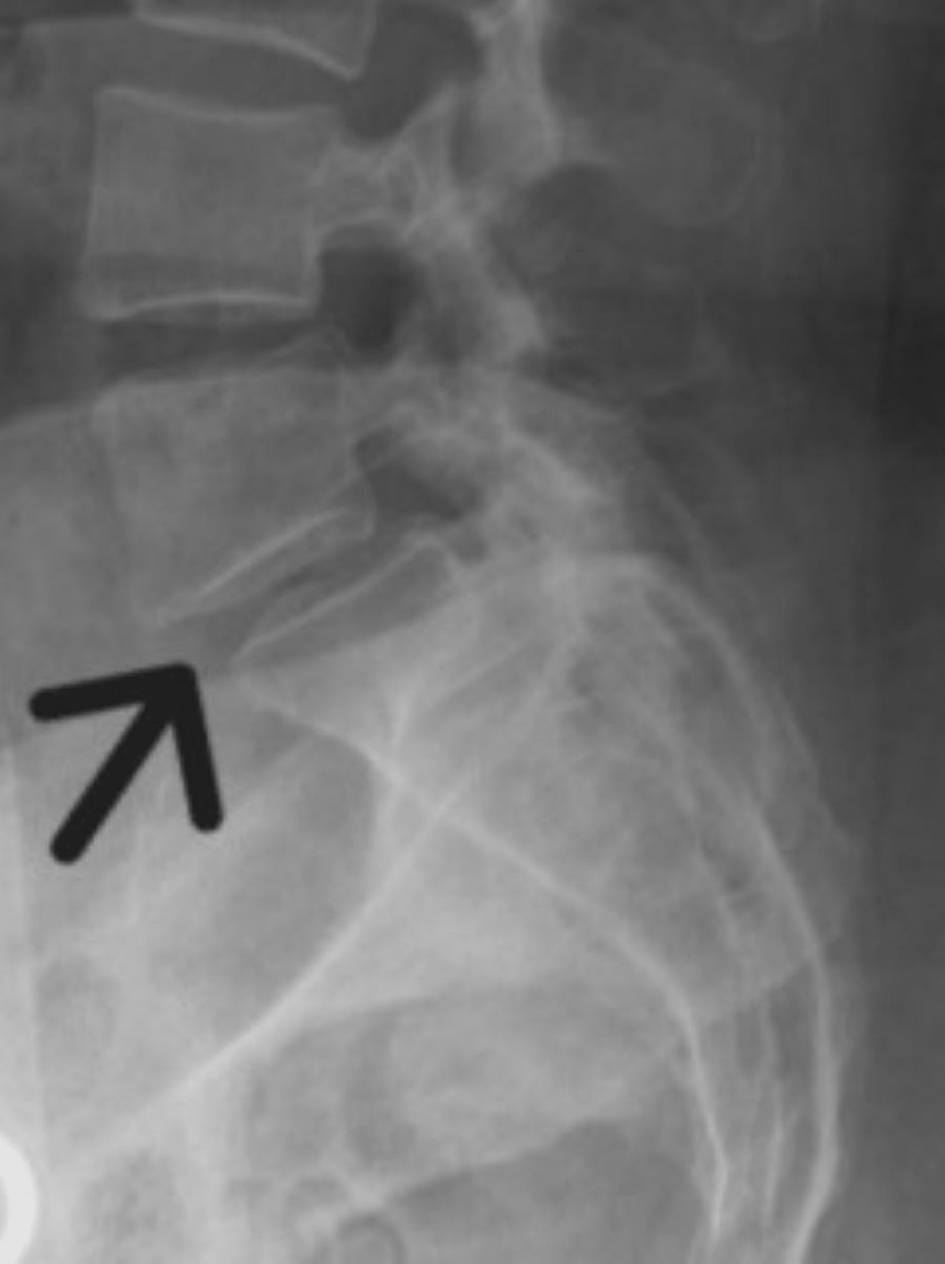
Anterolisthesis in between fifth lumbar vertebrae and the first sacral vertebrae (arrow).

**Figure 2:**
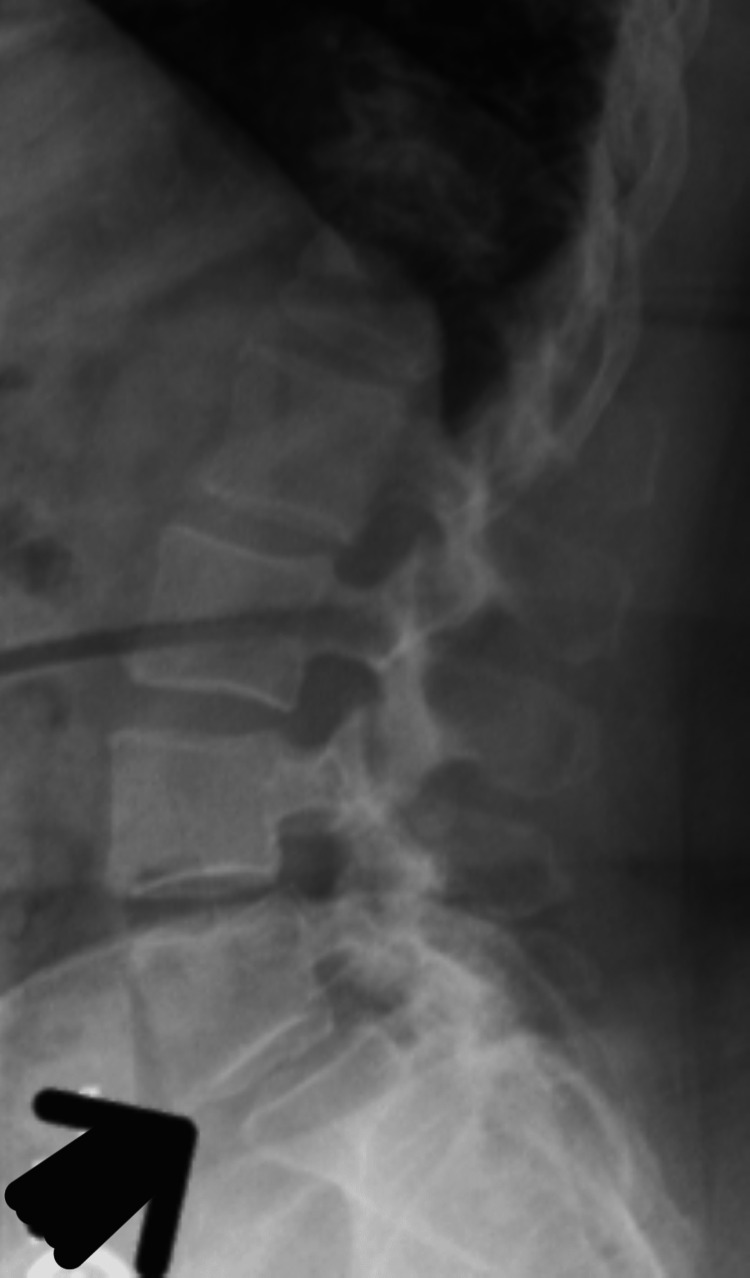
The x-ray image of the lumbosacral region. The image shows multilevel degenerative changes in the fourth lumbar to the first sacral vertebrae and a grade 1 anterolisthesis between the fifth lumbar vertebrae and the first sacral vertebrae (big arrow).

**Figure 3:**
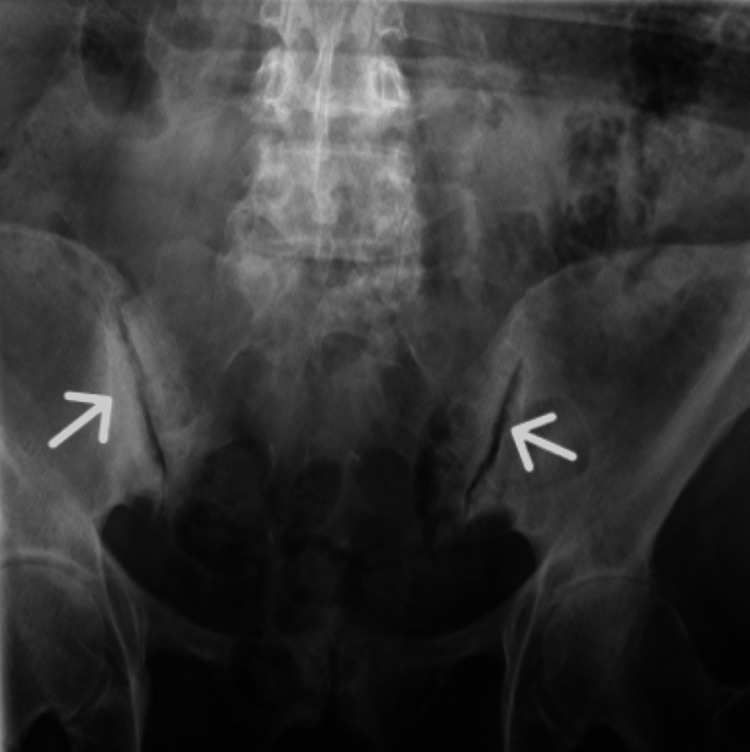
The x-ray image of the sacroiliac joints. The image shows marked bilateral sclerosis around the sacroiliac joints, especially on the iliac bones (arrows) without any evidence of inflammation (such as erosions and narrowing) within the sacroiliac joint spaces (diagnostic of osteitis condensans illi).

An imaging x-ray of the lumbar spine showed multilevel degenerative changes of the fourth lumbar vertebrae to first sacral vertebrae and grade 1 anterolisthesis of the fifth lumbar vertebrae to first sacral vertebrae (Figures 1, 2). In addition, the x-ray of the sacroiliac joint showed marked sclerosis of the bilateral sacroiliac (SI) joints, with the right greater than left without discrete erosions (Figure 3).

The patient was managed conservatively with NSAIDs and physical therapy. After three months, she finally reported to the clinic, saying that her back pain had subsided.

## Discussion

The prevalence of osteitis condensans ilii (OCI) in the general population is between 0.9% and 2.5% [4]. However, in those patients who are explicitly undergoing imaging studies and evaluation for inflammatory arthropathy, the prevalence becomes as high as 8.9% [4]. Osteitis condensans ilii is often found in pregnant women, puerperium or women of childbearing age [5]. Nevertheless, although rare, as evidenced in this study, it is possible to find this condition in patients outside the defined age for conception. However, it is difficult to tell precisely when this disease starts in a postmenopausal woman with compounding incidental imaging findings of a degenerative low-grade spondylolisthesis which is usually asymptomatic in most cases [6,7]. As observed in our case presentation, we believe several factors contributed to why this condition was present in our patient. These include progressive weight gain, childhood trauma history, and increased regular activities. In a study done by Shipp and Haggart in 1950, they showed that chronic low-back pain was commonly reported in patients with osteitis condensans ilii. They explained that lower back pain symptoms in OCI were exacerbated by activity and relieved by rest in almost all cases. They also reported that of the 100 patients in their clinical study, about 48 individuals identified their pregnancy as the onset of their symptoms. In contrast, others reported the onset of symptoms following rapid weight gain or excessive physical fatigue. Shipp and Haggart also reported that 64 patients in their clinical study were overweight [8]. The patient in this study had a childhood history of trauma and was also obese. Therefore, it could be argued that the extra weight around her pelvis may have contributed to the mechanical stress on her sacroiliac joint leading to sclerosis.

Osteitis condensans ilii (OCI) has been described as a benign condition usually managed conservatively with physical therapy and non-steroidal anti-inflammatory medications. Anti-inflammatory medications such as steroids and anesthetic injections have been utilized in treating OCI, even though it is not an outright inflammatory condition. However, there appears to be a subset of patients who develop a resistant form of this disease, necessitating a different approach to the treatment principles of the condition. Sacroiliac arthrodesis is a surgical procedure proposed for resistant cases of osteitis condensans ilii. However, in addition to being invasive, this procedure leads to negative long-term results and multiple complications.

Lee et al. suggested surgical decompression in one refractory case of OCI [9]. Similarly, another study by Ayoub et al. proposed a favorable outcome using a minimally invasive method to treat chronic osteitis condensans ilii. Their approach involved decompressing the sclerotic sacroiliac joints with percutaneous cannulated drills and using the Bath Ankylosing Spondylitis Functional Index (BASFI) for functional outcome evaluation. Although not widely accepted, the approach in their study showed a mean BASFI score improvement from 3.7±0.6 preoperatively to 1.3±0.2 during follow-up, with a statistical significance of p≤0.001. In addition, they reported no relapse cases or significant complications following their minimally invasive procedure [10]. Though OCI symptoms are self-limiting and even the classical radiological findings can disappear with time, it is essential to diagnose OCI in a timely fashion as refractory cases can cause varying degrees of disabilities, resistant to medical therapy, and necessitate a surgical intervention [5].

## Conclusions

Chronic back pain is a common complaint for ambulatory clinic patients. Therefore, when creating a differential diagnosis for patients complaining of back pain, the primary care physician and clinicians need to have a high index of suspicion for identifying and treating uncommon cases of back pain, such as oseitis condensans ilii. This study shows that the diagnosis is straightforward and can be made with simple x-ray films of the sacroiliac joints. Also, a prompt diagnosis can alleviate the need for unnecessary tests, medications, and misdiagnoses.
